# Two scientific perspectives on nerve signal propagation: how incompatible approaches jointly promote progress in explanatory understanding

**DOI:** 10.1007/s40656-024-00644-4

**Published:** 2024-11-21

**Authors:** Linda Holland, Henk W. de Regt, Benjamin Drukarch

**Affiliations:** 1 Amsterdam Neuroscience, Department of Anatomy and Neurosciences, Vrije Universiteit Amsterdam, Amsterdam UMC, Amsterdam, The Netherlands; 2 Institute for Science in Society (ISiS), Radboud University, P.O. Box 9010 (postvak 77), 6500 GL Nijmegen, The Netherlands

**Keywords:** Scientific perspectivism, Scientific understanding, Progress in explanatory understanding, Philosophy of science in practice, Nerve signal propagation, Hodgkin–Huxley model

## Abstract

We present a case study of two scientific perspectives on the phenomenon of nerve signal propagation, a bio-electric and a thermodynamic perspective, and compare this case with two accounts of scientific perspectivism: those of Michela Massimi and Juha Saatsi, respectively. We demonstrate that the interaction between the bio-electric perspective and the thermodynamic perspective can be captured in Saatsi’s terms of progress in explanatory understanding. Using insights from our case study, we argue that both the epistemic and pragmatic dimensions of scientific understanding are important for increasing explanatory understanding of phenomena. The epistemic dimension of understanding is important for increasing the number of *actually* answered what-if-things-had-been-different questions about a phenomenon, the pragmatic dimension for pointing out the *potentially* answerable what-if questions that have been overlooked or purposefully neglected thus far. Exposing the limitations of the acquired understanding within a particular perspective can be achieved by criticizing the assumptions that have been adopted to make models of the perspective intelligible, but that are considered problematic from a rival perspective.

## Introduction

With the work of Ronald Giere (2006) and more recently Michela Massimi (2018, 2022) scientific perspectivism—or perspectival realism—has become a notable position in the philosophy of science. Perspectivism acknowledges that science is historically and culturally situated and thereby always practiced from a particular perspective. These perspectives may change in the course of time and can also vary across communities. Moreover, there is no way to establish that one perspective is true while the others are false: different, even contradictory, perspectives may all be valid and valuable. Still, both Giere and Massimi claim that this does not necessarily lead to relativism and/or anti-realism, and that perspectivism can be formulated as a sophisticated form of realism.

In our paper we examine the value of scientific perspectivism, in two different appearances, for analyzing the practice of science. More concretely, we present a detailed case study of a controversy in neuroscience that may be interpreted as a mismatch between different perspectives on the phenomenon of nerve signal propagation. For the purpose of introducing these perspectives, we will define a perspective broadly as the set of claims considered central to explaining the phenomena that are studied by a scientific community that is historically and intellectually situated. The perspectives involved in the controversy about nerve signal propagation are, on the one hand, the bio-electric perspective—which has given rise to the well-known Hodgkin–Huxley model (1952d)—and, on the other hand, the more recent thermodynamic perspective (Andersen, 2009; Schneider, 2021)—which has inspired alternative models of nerve signal propagation.

After introducing the bio-electric and thermodynamic perspectives using the rather broad definition of perspectivism above, we will examine whether and how two philosophically fleshed-out accounts of perspectivism—the accounts of Massimi and Saatsi—can deal with this case of scientific practice. Our analysis shows that perspectivism is indeed a fruitful approach for the interpretation of this controversy. It turns out, however, that the value of a perspectivist analysis does not so much regard questions concerning the truth of the theories and models offered in the different perspectives, but rather the question of how the different perspectives stimulate progress in explanatory understanding.

This result accords with a recent proposal by Saatsi (2019), who has developed a perspectivist account of science in terms of explanatory understanding. According to this account, explanatory understanding is increased when scientists are able to answer more what-if-things-had-been-different questions about a phenomenon. Thus, the added value of different perspectives on the same phenomenon consists either in the ability to provide answers to more what-if questions about a phenomenon or in making the existing explanations of the phenomenon more cognitively salient for scientists, due to the use of different models in different perspectives. Saatsi considers the former way of increasing explanatory understanding, the way related to the epistemic dimension of understanding, particularly important for the realist.

Yet, our case study suggests that the added value of different perspectives for progress in explanatory understanding also can involve something else that is important for the realist: exposing and criticizing the limitations of the understanding obtained in rival perspectives as a result of adopted assumptions to make perspectival models intelligible for scientists. Although criticizing these limitations does not directly lead to an increase in explanatory understanding by *actually* being able to answer more what-if questions about the phenomenon, it does show what *potentially* answerable what-if questions about the phenomenon have been overlooked or purposefully neglected in the past due to assumptions that have been made to make models intelligible. Addressing these what-if questions may increase explanatory understanding of the phenomenon in the future. This entails that also the pragmatic dimension of understanding is important for the perspectival realist.

The outline of our paper is as follows. In Sect. 2 we introduce the bio-electric perspective and the thermodynamic perspective on nerve signal propagation. In Sect. 3 we discuss two different accounts of scientific perspectivism: the accounts of Massimi and Saatsi. In Sect. 4 we discuss these philosophical accounts in relation to our case study, showing on which points they are in (dis)agreement with each other. We conclude our paper in Sect. 5.

## The study of nerve signal propagation within different scientific communities

### The bio-electric perspective on nerve signal propagation

The bio-electric perspective on the phenomenon of nerve signal propagation is considered central to explaining this phenomenon by a scientific community that has studied bio-electrical phenomena in general, and the nerve signal in particular, for approximately two centuries. The work of Luigi Galvani in 1791 is regarded as one of the cornerstones of this study. He showed that muscle contractions are due to ‘animal electricity’. After his discovery, a lot of theoretical and experimental work has been done to examine the nature of this animal electricity and its role in the physiological process of nerve signal propagation (for a historical overview, see Drukarch et al. (2018)).

One of the models that was developed in this context is the well-known 1952 Hodgkin–Huxley model (HH-model) of the action potential (i.e., the electrical manifestation of the nerve signal). The HH-model (Hodgkin & Huxley, 1952d) is a mathematical model that proved important in the development of neurophysiological theorizing and experimenting. The model is based on a series of experiments on the giant axon of the squid (Hodgkin & Huxley, 1952a, b, c; Hodgkin et al., 1952). In these experiments the voltage clamp technique was used. With this technique, the potential over the membrane of an isolated axon can be changed suddenly, after which it is held constant (clamped) at a specific membrane potential using an electrical feedback circuit. In these experiments it is assumed that the current that must be injected in the nerve fiber to keep the membrane potential constant is similar to the current that flows through the membrane (Hodgkin et al., 1952).

In the HH-model the neural membrane is modeled as an electric circuit consisting of a capacitor, three resistors and three batteries (Hodgkin & Huxley, 1952d). Hodgkin and Huxley used Ohm’s law and Kirchhoff’s laws to develop a mathematical equation describing the total current in this circuit. The total membrane current ($I$; see Eq. 1) consists of a capacity current [1] involving a change in the ion density at both sides of the neural membrane and an ionic current [2–4] due to ions flowing through the membrane (Hodgkin et al., 1952) upon depolarization of the membrane (i.e., a change in membrane potential above the required threshold to generate an action potential). The variables in Eq. 1 are described in more detail in Box 1.

**Box 1:** Detailed description of Eq. 1

The capacity current [1] depends on the membrane capacitance (${C}_{M}$) and the change in the displacement of the membrane potential from it resting value over time $\left( {\frac{{{\text{d}}V}}{{{\text{d}}t}}} \right)$. The ionic current is further divided into a potassium ion ($K$) current [2], a sodium ion ($Na$) current [3] and a leakage current of other ions ($L$) [4]. Each of the ionic currents depends on the permeability of the neural membrane for the respective ion species and is described in terms of an ionic conductance (${g}_{ion}$), which is the inverse of the electrical resistance, and a driving force, which is the result of the difference between the displacement of the membrane potential from its resting value ($V$) and the equilibrium potential for the ion given as a displacement from the resting membrane potential (${V}_{ion}$).	

1 $$I=\left[1\right] {C}_{M}\frac{dV}{dt}+\left[2\right] {g}_{K}\left(V-{V}_{K}\right)+\left[3\right] {g}_{Na}\left(V-{V}_{Na}\right)+\left[4\right] {g}_{L}\left(V-{V}_{L}\right)$$

In the HH-model (Hodgkin & Huxley, 1952d; Hodgkin et al., 1952), it is assumed that the membrane capacitance is constant.^1^ This assumption plays an important role in the controversy about nerve signal propagation between the communities putting forward the bio-electric perspective and the thermodynamic perspective. It is justified by the measured time course of the capacity current in the voltage clamp. As a result of this assumption, the capacity current only plays a role in the quantitative description when the membrane potential of the isolated axon is suddenly changed in the beginning of a voltage clamp experiment. It will become zero when the membrane potential is kept constant during the rest of the experiment. As a consequence of this assumption, the current that is measured in the experiment while the membrane potential is kept constant can be interpreted as ionic current, and these measurements can be used as input for the model. The equations (of which Eq. 1 is the main equation) that Hodgkin and Huxley (1952d) developed based on their experiments capture key characteristics of action potentials measured in experiments, e.g., the form (the measured change of voltage over time), amplitude and threshold of action potentials.

Although Hodgkin and Huxley had experimentally established that the membrane becomes selectively permeable for a specific ion species when the potential across the membrane changes, they did not know *how* these ions cross the membrane (Hodgkin & Huxley, 1952d). For this reason, they constructed equations for the sodium and potassium conductance terms in Eq. 1 by fitting them to experimental data. They assumed that these conductances were functions of voltage and time and introduced several ad hoc parameters to obtain equations that “describe the conductances with reasonable accuracy and are sufficiently simple for theoretical calculation of the action potential” (Hodgkin & Huxley, 1952d, p. 506).

Following the introduction of the HH-model, much research effort was put into establishing *how* ions cross the neural membrane. To illustrate this, we focus on the scientific study of the sodium ion channel.^2^ To study the sodium currents through the neural membrane in more detail a new technique needed to be developed: the patch clamp technique (Neher & Sakmann, 1976). With this technique (a refinement of the voltage clamp technique), currents through small patches of the neural membrane can be measured. The characteristics of the ‘macroscopic’ sodium current measured with the voltage clamp technique were used as a constraint for the ‘microscopic’ sodium current measured with the patch clamp technique. Assuming that the measured microscopic currents are the result of identical, independently functioning sodium ion channels, it was indeed shown that the characteristics of the average of the sum of multiple microscopic currents are in accordance with those of the macroscopic current (Sigworth & Neher, 1980).

For further characterization of the sodium ion channel, its molecular structure was studied as well. First, it was established that the biophysical properties of the protein that was expected to be the sodium ion channel were in accordance with the properties of the sodium ion channel as defined on the basis of patch clamp measurements (Rosenberg et al., 1984). After this, the genetic code of this protein was identified and the corresponding amino acid sequence was determined. Informed by this, several models of the protein structure of the sodium ion channel were put forward, amongst them the models of Noda et al. (1984) and Guy & Seetharamulu (1986). These models were then used to study sodium channel activation and inactivation by changing the molecular structure of the sodium ion channel and investigating the resulting electrophysiological characteristics (Stühmer et al., 1989). In this way, by studying activation and inactivation of ion channels during the action potential, scientists developed their explanation of the action potential further.

The current textbook explanation of the action potential goes roughly as follows (e.g., Purves et al., (2008)). Upon depolarization of the membrane, selective voltage-gated sodium ion channels open, allowing sodium ions to diffuse into the nerve cell due to their electrochemical gradient. After that, the sodium ion channels transition to an inactive state ending the diffusion of sodium ions. At the same time as the inactivation of sodium ion channels, selective voltage-gated potassium ions channels open, allowing potassium ions to diffuse out of the nerve cell due to their electrochemical gradient. Since the potassium ion channels return to their closed state slowly, the membrane hyperpolarizes (the membrane potential becomes lower than the resting membrane potential). After the potassium ion channels close, the resting membrane potential is restored due to other membrane protein pumps that actively move ions into or out of the nerve cell.

Thus far, we have solely focused on the action potential that can be measured with the voltage clamp, in which the whole isolated axon is stimulated at once with an electrode. However, scientists are ultimately interested in explaining action potential *propagation* (i.e., an action potential that travels from one end of a nerve fiber to the other after stimulation of a local part of the membrane), since this is considered the basis of neural communication in the nervous system. For this, the above explanation does not suffice, since it only explains the flow of ions *across* the membrane and not the propagation of the action potential *along* the axon. Using cable theory and assuming that the action potential travels at constant speed independent of voltage (Appali et al., 2012), Hodgkin & Huxley (1952d) developed Eq. 1 further into a differential equation that describes the currents during action potential *propagation*:

2 $$\frac{a}{2R{\theta }^{2}}\frac{{d}^{2}V}{d{t}^{2}}={C}_{M}\frac{dV}{dt}+{g}_{K}\left(V-{V}_{K}\right)+ {g}_{Na}\left(V-{V}_{Na}\right)+ {g}_{L}\left(V-{V}_{L}\right)$$

In this equation, $a$ is the radius of the axon, $R$ is the resistance of the axoplasm, $\theta$ the velocity of conduction, and $\frac{{d}^{2}V}{d{t}^{2}}$ the rate at which the change in voltage is changing over time. Notice that the membrane capacitance is still assumed constant in this equation. Modelling the propagating action potential with this equation, Hodgkin & Huxley (1952d) succeeded in capturing key characteristics of the measured *propagating* action potentials in the squid axon (e.g., form, amplitude and velocity).

On the basis of the performed research, the current textbook explanation exemplifying the bio-electric perspective on action potential *propagation* goes as follows (e.g., Purves et al. (2008)): some of the local current in the axon generated by the inward flow of sodium ions during the action potential spreads passively along the axon (until the current leaks out of the axon through the membrane), depolarizing the membrane at a neighboring part of the axon and opening sodium ion channels there. The addition of passive flow along the axon to the explanation is based on studies of passive current flow in axons upon subthreshold stimulation of the neural membrane (thus, without generating an action potential) (Hodgkin & Rushton, 1946; Rall, 1977). However, within the context of the discussion following hereafter, it is important to note that unequivocal experimental evidence that current spreads passively during action potential propagation is lacking. This means that, despite the current textbook HH-model, there is still a gap in the explanation of action potential *propagation*. Scientists developing a thermodynamic perspective on nerve signal propagation have criticized this bio-electric perspective on the phenomenon, in particular the assumption that the membrane capacitance is constant in the HH-model. They claim that although this assumption is justified in the context of the voltage clamp, it is not in the context in which nerve signal *propagation* is studied. We will explain this in detail in the next section.

### The thermodynamic perspective on nerve signal propagation

The challenge of explaining *how* ions traveled across the neural membrane, which was solved by the community developing the bio-electric perspective on nerve signal propagation, was not the only challenge that was presented by the HH-model. There was experimental evidence available, for instance regarding temperature (Hill, 1912) and mechanical (Hill, 1950) changes measured during nerve signal propagation, that could not be accounted for by the HH-framework. In fact, Hodgkin (1964, p. 70) himself points out that “[i]n thinking about the physical basis of the action potential perhaps the most important thing to do at the present moment is to consider whether there are any unexplained observations which have been neglected in an attempt to make the experiments fit into a tidy pattern”. Within this context, he discusses in particular the temperature changes measured during action potential propagation as the most puzzling observation made.

Many studies performed in the second half of the twentieth century have confirmed that action potential propagation coincides with (largely) reversible thermal changes: heat is released and subsequently (partially) reabsorbed by the nerve cell as the nerve signal passes by (e.g. Howarth et al. (1968); Tasaki & Byrne (1992); Tasaki et al. (1989)). This heat release and subsequent reabsorption cannot be explained with the help of the HH-model (Hodgkin, 1964; Howarth et al., 1968). In addition, several studies have also confirmed that action potential propagation is associated with mechanical changes: swelling and subsequent shrinking, and shortening of the nerve cell (e.g., Iwasa & Tasaki (1980); Tasaki & Iwasa (1982); Tasaki et al. (1989)). These mechanical manifestations of the nerve signal are not taken into account by the HH-model but are not necessarily in disagreement with the model.

Recently, a group of membrane biophysicists has criticized the HH-model for being “incapable of explaining or predicting many experimentally observed characteristics of nerve signal propagation” (Andersen et al., 2009, p. 107), thereby pointing to the discussed experimental evidence already gathered in the second half of the twentieth century. This group of membrane biophysicists takes a radically different approach to studying nerve signal propagation. More in particular, to reach their goal of explaining nerve signal propagation, they strive to develop a thermodynamic theory of this phenomenon. In their development of this thermodynamic perspective on nerve signal propagation, the work from theoretical physicist Kaufmann in the late 1980s plays a central role. Kaufmann applied Einstein’s thermodynamic approach to living systems and used it to develop a new theory of nerve signal propagation (Kaufmann, 1989).

Following Einstein’s approach to thermodynamics,^3^ the scientists start by empirically describing the thermal behavior of the thermodynamic system of interest in terms of compressibility, heat capacity, conductivity, etc. Using the second law of thermodynamics, the entropy values for different states of the thermodynamic system are calculated. Next, Boltzmann’s principle is used to calculate the statistical probability of these individual states. A benefit of this approach is that one does not have to assume anything about the structure of the thermodynamic system of interest to determine the probability of the system’s states and ultimately the thermodynamic properties of the system. Rather, one starts with the description of the empirically accessible thermal behavior of the system to derive probable thermodynamic states and properties that are purely phenomenologically defined (Schneider, 2021).

The thermodynamic system that has been identified by these scientists as the system of interest is the neural membrane interface, which consists of lipids and proteins, but also ions and water, etc. Since this interface is to some extent decoupled from its surroundings, it has its own thermodynamic states and properties (Fillafer et al., 2021; Heimburg & Jackson, 2005). Experimental support for this thermodynamic system comes from a study by Terakawa & Nakayama (1985), which demonstrates that nerve signals can still be excited in axons after the removal of intracellular material, indicating that nerve signals propagate in the neural membrane.

The experimental results concerning the electrical, mechanical and thermal aspects of nerve signal propagation in the twentieth century suggest that this phenomenon has a quasi-adiabatic character: there is no or very little heat transfer between the nerve and its surroundings. This has inspired the idea that the nerve signal can be modeled as an acoustic pulse. For such a pulse, all the reported aspects of nerve signal propagation (e.g., electrical, mechanical and thermal) follow from the second law of thermodynamics (using the Maxwell relations). Since the membrane interface is to some extent decoupled from its surroundings, propagation of an acoustic pulse follows from momentum conservation. Thus, an acoustic pulse in the membrane interface can be approached as a propagating thermodynamic state change, which can be studied by measuring pressure, temperature, volume, electric fields, pH, etc. during propagation (Schneider, 2021).

More specifically, nerve signals are considered to be local *nonlinear* acoustic pulses due to a thermodynamic transition of the membrane interface. Biological membranes are in a fluid phase under physiological conditions. Upon changing experimental circumstances like temperature, pressure or pH value, the state of the membrane can switch to a slightly denser gel phase (Fillafer et al., 2005; Schneider, 2021). This density change during acoustic pulse propagation in biological membranes can, for instance, explain the measured swelling and subsequent shrinking, and shortening of the nerve cell during nerve signal propagation (e.g., Iwasa & Tasaki (1980); Tasaki & Iwasa (1982); Tasaki et al., (1989)).

Combining the evidence concerning mechanical changes in the nerve cell during nerve signal propagation and the evidence that nerve signals are able to propagate in nerve cells without a cytoplasm, it is inferred in the thermodynamic perspective that it is the neural membrane and its surrounding structures (i.e., the membrane interface) that is swelling and shrinking during nerve signal propagation. This conclusion has the important consequence that the assumption in the HH-model that the membrane capacitance is constant is not justified in the context of nerve signal propagation.^4^ Moreover, the scientists developing the thermodynamic perspective on nerve signal propagation point out that the density change during acoustic pulse propagation is able to account for the measured voltage pulse during nerve signal propagation in terms of a changing membrane capacitance (rather than ionic currents across and passive currents along the neural membrane) (Andersen et al., 2009).

During acoustic pulse propagation heat is released when the membrane transitions from fluid to gel phase and is reabsorbed when the membrane transitions back to the fluid phase. In experiments it has indeed been shown that heat is released and subsequently partially reabsorbed during nerve signal propagation (Howarth et al., 1968; Tasaki & Byrne, 1992; Tasaki et al., 1989). The thermodynamic theory explains the heat produced during nerve signal propagation as the result of a reversible thermodynamic process. By contrast, the bio-electric perspective—that cannot account for the temperature changes during nerve signal propagation—implies a thermodynamic irreversible process (Drukarch et al., 2022; Heimburg, 2021).

Thus, the thermodynamic and bio-electric perspectives on nerve signal propagation are incompatible. The thermodynamic perspective does *not* assume the membrane capacitance constant and models nerve signal propagation as a *reversible* thermodynamic process, whereas the bio-electric perspective *does* assume the membrane capacitance constant and models nerve signal propagation as a thermodynamic *irreversible* process. But while the thermodynamic perspective can quantitatively account for the temperature changes measured during nerve signal propagation, the bio-electric perspective cannot.^5^ Moreover, the thermodynamic explanation provides a direction to explore why the bio-electric perspective cannot account for these temperature changes: the influence of membrane capacitance during nerve signal propagation.

## Perspectivism in the philosophy of science

Above we have discussed two different perspectives on nerve signal propagation. However, we have used a very broad definition of a scientific perspective: the set of claims considered central to explaining the phenomena that are studied by a scientific community that is historically and intellectually situated. In the present section we will review the current debate on perspectivism in the philosophy of science by discussing and comparing two different accounts that both fit this definition. In Sect. 4 we will apply them to our case study and evaluate which account is most suitable for analyzing the case.

While perspectivism has a long-standing history in philosophy, with Kant and Nietzsche as pivotal figures, use of the term has become popular in philosophy of science only recently. It was Ronald Giere who, in his book *Scientific Perspectivism* (2006), first developed a perspectivist account of scientific knowledge. By drawing an analogy between human color vision—which is produced and limited by the interaction between observer and the object observed—and scientific knowledge production, Giere argues that scientific knowledge is produced and limited by the measuring instruments and theoretical models that are employed in a scientific community. Giere’s perspectival realism is built on his well-known analysis of scientific modeling, according to which models are derived from general scientific principles and are intended to represent specific aspects of the world to a certain degree (Giere, 2004). He argues that the fit between models and the world can better be characterized in terms of similarity than truth, since a perfect fit between model and world is not to be expected.

Giere distinguishes two ways in which scientific knowledge can be perspectival: it can be part of an instrumental perspective and/or a theoretical perspective. In the case of an instrumental perspective, scientists first have to choose the scientific instruments they use to study the world. Thus, they see the world ‘through’ these instruments. Accordingly, scientists should take into account that the conclusions that they draw based on observation or measurement with their instruments are always relative to these instruments. For theoretical perspectives, his argument that scientific knowledge is perspectival goes along similar lines. Scientists first have to accept specific theoretical principles. Based on these principles, models are developed that represent the world. So, what aspects of the world are represented in models and to what degree depends ultimately on the theorical principles that have been accepted by scientists. As a result, the conclusions that are drawn about the world are relative to a theoretical perspective.

Although Giere prefers to talk about similarity between models and the world rather than about truth, he does spend a few words on truth in his book. We will address this point briefly, since Massimi criticizes Giere’s account based on an analysis of his use of the term truth. According to his perspectivist view, truth cannot be understood in objectivist terms, rather “claims about the truth of scientific statements or the fit of models to the world are made […] within perspectives” (Giere, 2006, p. 82) and “truth claims are always relative to a perspective” (Giere, 2006, p. 81). Here again, he stresses that one first has to choose which scientific instruments and theoretical principles to use. After that, one makes claims that can be judged true or false relative to these choices.

Giere (2006) intended his perspectivism to support a “perspectival rather than objectivist understanding of scientific realism” (p. 6) that “does not degenerate into a silly relativism” (p. 13). Massimi (2017) is worried, however, that his analysis of truth brings Giere’s account too close to relativism. If the truth of a scientific claim is relative to a scientific perspective, it becomes impossible to compare claims across perspectives. What is true in one perspective can be false in another. In response to this worry, Massimi (2018) develops a notion of cross-perspectival truth.

Before introducing her notion of cross-perspectival truth, we need to clarify what Massimi means with a scientific perspective. In line with the broad definition that we used to introduce the bio-electric and thermodynamic perspectives on nerve signal propagation, Massimi (2018, 2022) refers with the term scientific perspective to a historically- and intellectually-situated scientific practice. She focuses primarily on the knowledge claims that are put forward by a scientific community. These claims have to be reliably produced with the help of theoretical, experimental and technological resources that are available to the scientific community and have to be justified using second-order (methodological-epistemic) claims.

At the heart of Massimi’s account lies a distinction between two different roles for scientific perspectives: they can provide a *context of use* and a *context of assessment* (Massimi, 2017, 2022). In the context of use new knowledge claims are advanced. This is in line with the traditional characterization of the role of a scientific perspective. But, according to Massimi, perspectives can provide a context of assessment as well. Scientists in a context of assessment can offer a different standpoint from which knowledge claims advanced in the context of use can be evaluated.

In the *context of use* certain ‘standards of performance adequacy’ are established. Examples of such standards are: accuracy in relation to fundamental equations, empirical testability within the limits of well-defined tests, and heuristic fruitfulness across a variety of practices. The truth or falsity of knowledge claims is determined based on these standards of performance adequacy. However, the standards of performance adequacy that are adopted in the context of use are necessary but not sufficient to establish whether a scientific claim is also cross-perspectivally true. Although a scientific claim can meet the standards of performance adequacy within a particular scientific perspective, it is still possible that a scientific claim is true in one perspective and false in another.

This is the reason why Massimi introduces a second role for scientific perspectives: a scientific perspective can provide a *context of assessment* in which it is evaluated whether a scientific claim that is advanced in the context of use of another perspective is also cross-perspectivally true. For this evaluation, additional information, for example further experimental evidence, is used in order to determine whether the knowledge claim continues to satisfy the adopted standards of performance adequacy it was meant to satisfy. If the knowledge claim continues to satisfy these standards, the claim can be considered cross-perspectivally true.

Massimi supports her account with examples from scientific practice, covering both diachronically and synchronically different perspectives. Of these, we will consider the most extensively discussed one concerning synchronically different perspectives: hydrodynamics and statistical mechanics (discussed in Massimi (2017 and 2018)). In hydrodynamics the knowledge claim ‘water is a liquid with viscosity’ is put forward based on standards of performance adequacy such as: its accuracy with regard to the Navier–Stokes equations and its empirical testability in measurements. Although viscosity is not a primitive property of water within the perspective of statistical mechanics, the truth of the above knowledge claim can still be assessed and confirmed from the statistical-mechanical perspective by deriving the viscosity of water from the statistical properties of molecules’ mean flow using various approaches. Thus, the additional information from statistical mechanics confirms that the knowledge claim produced in hydrodynamics meets its original standards of performance adequacy, and thus can be considered cross-perspectivally true.

Consequently, Massimi’s account implies that interaction between perspectives is required for the ascription of cross-perspectival truth to knowledge claims. Her recognition that interaction between perspectives is important has been a key contribution to the debate on perspectivism. However, we will suggest, based on our case study, that this interaction can be better captured in terms of explanatory understanding than cross-perspectival truth.

Saatsi (2019) is the philosopher who has introduced the notion of explanatory understanding in the debate on perspectivism. He agrees with Massimi that scientific realism should accommodate the fact that scientists may study the world from different perspectives. However, rather than focusing on scientific knowledge as the output of our perspectival scientific study of the world, he focuses on scientific understanding, which he regards as essentially different from knowledge: “understanding […] is not knowledge but rather an ability” (Saatsi, 2019, p. 66).^6^ More specifically, he develops a notion of *explanatory* perspectives that he defines as follows: “ways of thinking about and representing a subject matter (say, light) in an explanatory context, which function to augment our understanding of the natural phenomena we are theorizing about (say, the rainbow)” (Saatsi, 2019, p. 66).

Saatsi provides a basic factivity requirement for explanatory perspectives by introducing a counterfactual-dependence account of explanation (among others defended by Woodward (2003)). Explanatory counterfactuals provide information that answers what-if-things-had-been-different questions: how would the explanandum have been different, had the explanans been different? According to Saatsi, the relation between explanans and explanandum is an objective, explanatorily relevant, worldly dependency relation. An explanation is explanatory and factive to the extent that it correctly captures this dependence. Yet, a counterfactual explanation should also stand in such a cognitive relationship to human beings that humans *understand* the explanation. It is here that, according to Saatsi, the non-factive aspects of theoretical representations come into play. The reason is that whether or not human scientists are *actually* able to answer what-if-things-had-been-different questions depends in part on pragmatic factors such as their education and practical experience, as well as on the general limits of human cognition. In other words, scientific understanding has a crucial pragmatic dimension and is, more than explanation, “a matter of skillful agency” (Leonelli, 2009, p. 197). A consequence of this is that scientific understanding is typically promoted by theories or models that are intelligible rather than veridical. In Saatsi’s words: user-friendliness trumps fidelity. This is often achieved by virtue of idealizations, simplifications, or outright misrepresentation that theoretical representations like scientific models become intelligible and can be employed to understand the phenomena (de Regt, 2017).

Thus, on Saatsi’s account the goal of explaining is to provide understanding, which is an ability to answer a range of what-if-things-had-been-different questions about a certain explanandum. The more answers can be provided in this way, the higher the degree of the obtained explanatory understanding is. An increase in explanatory understanding can be achieved by capturing more and more worldly dependency relations (the factive aspect of scientific understanding) and/or by bringing the counterfactual explanations in a better cognitive relationship to scientists by idealizing, simplifying or misrepresenting the world (the non-factive aspect of scientific understanding). Scientists are not necessarily aware of what part of their explanatory perspective is factive and what part is non-factive.

Saatsi also discusses progress in explanatory understanding across different explanatory perspectives. Due to the fact that theoretical representations have non-factive aspects, they may give rise to incompatibilities between different explanatory perspectives that both provide understanding of a particular phenomenon. Which explanatory perspective is chosen from a set of incompatible perspectives as providing the best explanation of a phenomenon depends on contextual factors: it depends on the skills of the scientists who are studying the phenomenon which explanation is considered to be most cognitively transparent for them. Saatsi calls this the *pragmatic* dimension of understanding.

In order to argue that the history of science can be given a realist interpretation despite incompatible explanatory perspectives and different preferences of scientists for such perspectives, Saatsi focuses on the *epistemic* dimension of scientific understanding. According to him, progress in explanatory understanding across explanatory perspectives is achieved by the increasing ability of scientists to answer more and more what-if-things-had-been-different questions about a phenomenon, capturing “better and better how different explanandum variables depend on different explanans variables” (Saatsi, 2019, p. 76). Thus, understanding increases when new explanatory perspectives increase our ability to answer more what-if questions about a phenomenon that respond to worldly facts of dependency. This can be accomplished by introducing new explanatory variables that represent additional explanatory worldly dependencies and, less important for the realist, by providing explanations of already known explanatory dependencies in a cognitively more salient form for scientists.

In summary, on Saatsi’s account, the value of the interaction between perspectives is related to an increase in explanatory understanding. Explanatory perspectives can complement each other in this regard by answering more what-if questions about a phenomenon or by making the existing explanations of the phenomenon more cognitively salient for scientists. In the next section, we will demonstrate that our case study suggests that it is also fruitful to study how progress in explanatory understanding can be *promoted*. The pragmatic dimension of understanding gets here a more important role for the realist: by criticizing the possibly non-factive aspects of an explanatory perspective from another (incompatible) perspective, it can be shown how the explanatory understanding already obtained can be increased to capture worldly dependency-relations even better in the future.

## A perspectivist perspective on the controversy about nerve signal propagation

### Progress in explanatory understanding of nerve signal propagation within the bio-electric perspective

In this section, we will argue that progress in the bio-electric perspective on nerve signal propagation can be captured in terms of an increase in explanatory understanding of this phenomenon. In addition, we will compare the case study with the accounts of Saatsi and Massimi.

At the time that Hodgkin and Huxley were studying nerve signal propagation, the leading hypothesis was that the neural membrane was permeable for potassium ions in the resting state, leading to a membrane potential across the membrane (Bernstein, 1912). Bernstein assumed that the action potential was a result of a temporary degradation of the neural membrane that allowed all kinds of ions to diffuse across the membrane. Overton (1902) demonstrated, however, that sodium or lithium ions are required for the generation of an action potential, indicating that the permeability of the membrane is also ion-specific during action potential propagation. He was the first to suggest that the action potential was a result of the exchange of sodium and potassium ions across the membrane. Decades later, Hodgkin & Katz (1949) established that the amplitude of the action potential in the squid axons lowers as a function of the extracellular concentration of sodium ions. Based on this finding, they hypothesized that the membrane becomes selectively permeable for sodium ions at the peak of the action potential. To summarize, there were two main hypotheses at the time: the neural membrane is selectively permeable to potassium ions during rest and it becomes selectively permeable to sodium ions during the action potential.

However, experimental study of the changes in ion-specific permeability and resulting currents during action potential propagation was impossible due to the all-or-none behavior of action potentials (i.e., if the membrane potential reaches a given threshold, an action potential is generated). Stimulating the neural membrane above the threshold would lead to a spontaneous switch of the membrane from one to the other permeability state. The invention of the voltage clamp solved this problem, allowing the study of ionic currents during the action potential. This technique was used by Hodgkin and Huxley (in collaboration with Katz^7^) to study the time course of ionic currents during the action potential in isolated squid axons (Hodgkin & Huxley, 1952a, 1952b, 1952c; Hodgkin et al., 1952). In these experiments, they assumed that the current that must be injected in the nerve fiber to keep the membrane potential constant is similar to the current that flows through the membrane (Hodgkin et al., 1952). They demonstrated that decreasing (rather than increasing) the membrane potential induces an inward current followed by an outward current, showing that the ionic permeability of the neural membrane is voltage-dependent. Moreover, they eliminated the measured inward current by removing extracellular sodium in the experimental system and demonstrated that the efflux of introduced radioactive potassium ions in the axon closely correlates with the measured outward current (Hodgkin & Huxley, 1952b). The HH-model is based on the experiments with the voltage clamp and provides a quantitative description of the measured currents.

In the experimental approach of Hodgkin, Huxley and Katz we see clearly that they established a counterfactual dependence between the membrane potential and the influx of sodium ions in the nerve cell in the voltage clamp. Moreover, they established a counterfactual dependence between the membrane potential and an outward current. Although they could not establish counterfactually that this outward current consists of potassium ions, they came up with a method to correlate the two. Still, establishing these counterfactual dependencies and the correlation required the invention of the voltage clamp and the assumption that the current that must be injected in the nerve fiber to keep the membrane potential constant in the voltage clamp is similar to the current that flows through the membrane (and thus that the membrane capacitance is constant). Without this technique and assumption, the time course of ionic currents during the action potential could not be understood by scientists.

Although the experiments and model of Hodgkin, Huxley and Katz provided some explanatory understanding of nerve signal propagation, they were not able to counterfactually establish how ions cross the neural membrane during nerve signal propagation. Yet, the explanatory understanding that the ionic permeability of the neural membrane is voltage-dependent has guided the research to find voltage-gated ion channels that are selective for specific ion species. After finding and characterizing these channels, it became possible to answer even more what-if-things-had-been-different questions about nerve signal propagation. For instance, the question could be answered how the electrophysiological characteristics of the sodium ion channel changes if the molecular structure of the channel is changed (Stühmer et al., 1989).

Thus, the bio-electric perspective has clearly progressed due to an increase in the obtained explanatory understanding of nerve signal propagation by answering more and more what-if questions about this phenomenon. Yet, the consequences of the assumption that the membrane capacitance is constant in the HH-model has not been investigated, even though this assumption underlies the whole research program regarding the bio-electric perspective on nerve signal propagation. Accordingly, it is currently unknown whether or not this assumption is an idealization that leads to a misrepresentation of the world. Researchers involved in developing the bio-electric perspective nowadays are often not even aware of this assumption.

What is precisely the problem with this assumption? As already discussed in Sect. 2a, the assumption has been shown to be justified in the context of the voltage clamp. However, the conditions in nerve signal propagation experiments are quite different. In the voltage clamp an isolated axon is as a whole stimulated by an electrode, after which currents are measured. Accordingly, currents are measured at the point of stimulation. By contrast, in propagation experiments an isolated axon is locally stimulated, and the action potential is recorded some distance away from the stimulation side. Thus, in the HH-equations for the propagating action potential, currents measured at the point of stimulation are used to predict the membrane potential some distance away (rather than at the point of stimulation). However, it has not been studied whether and how the membrane capacitance changes during the propagating action potential. Therefore, it is not known to what extent the HH-model represents the conditions in the nerve cell a distance away from stimulation.

The HH-model does capture some key counterfactual dependencies of the propagating action potential: e.g., its form, amplitude and velocity. So, we can conclude that the model does provide a degree of explanatory understanding of the phenomenon of nerve signal propagation. But since the bio-electric perspective cannot account for the temperature changes during the propagating action potential, we also know that understanding of this phenomenon is not maximal and can be increased.

This analysis is in line with Saatsi’s account. What is acquired in a scientific perspective is explanatory understanding. This understanding is aimed at capturing worldly counterfactual dependencies. But these dependencies cannot be captured completely due to the non-factive aspects of the models used, which are required to make them intelligible for scientists. The empirical description of ionic conductances in the HH-model provides an example of this. Since Hodgkin and Huxley did not know how ions cross the neural membrane, they chose to describe the ionic permeability of the neural membrane by fitting it to experimental data with reasonable accuracy and sufficient simplicity to model ionic currents during the nerve signal. For this, they assumed that the permeability of the membrane is a function of voltage and time, and they had to introduce several ad hoc parameters. Explanatory understanding in the bio-electric perspective was increased when scientists were able to answer more what-if questions about the nerve signal after they identified voltage-gated ion channels as the molecular entities responsible for specific ion flows across the membrane.

As Saatsi points out, scientists are not always in the position to distinguish the factive and non-factive aspects of their explanatory perspectives. The assumption of a constant membrane capacitance in the HH-model that is both experimentally and theoretically required^8^ to be able to study the ionic currents during the nerve signal is a case in point. This assumption could be a non-factive aspect of the HH-framework in the study of nerve signal propagation. As scientists developing the thermodynamic perspective have pointed out: not only ionic currents across and along the neural membrane can account for the measured voltage pulse during nerve signal propagation, a capacity current due to a change in the membrane capacitance could account for this as well. As long as the contribution of a capacity current to nerve signal propagation is not investigated, it is not clear whether a constant membrane capacitance is a factive aspect or a non-factive aspect of the bio-electric perspective. If it turns out to be a *factive* aspect, this would imply that the worldly dependence between the neural membrane as described in the bio-electric perspective and the phenomenon of nerve signal propagation is rightly captured according to Saatsi’s standards. By contrast, if it turns out to be a non-factive aspect, this entails that—even though the bio-electric perspective provides explanatory understanding of nerve signal propagation—the propagating nerve signal is misrepresented (to a certain extent) in this perspective.

How well does Massimi’s account apply to the case study? The bio-electric perspective does not determine what is considered false and true by scientists that use this perspective as their starting point. In an interview with Douglas Fox for the popular-scientific magazine Scientific American, biophysicist Adrian Parsegian states: ““I don’t think anybody disputed that those things [i.e., the non-electrical manifestations of the nerve signal] were being seen, because [Tasaki, who studied these non-electrical manifestations extensively] was respected in the lab,” […] Rather Tasaki’s findings “were explained away as not central” to nerve signaling—nothing more than side effects of the voltage pulse. The underlying scientific questions “didn’t get resolved,” […] “One side got into the textbooks, and the other didn’t.”” (Fox, 2018, p. 64). Thus, scientists accepting the bio-electric perspective on nerve signal propagation do not question that the nerve signal has non-electrical manifestations, they simply do not think these manifestations are as important as the electrical ones. Even though the bio-electric perspective implies that the temperature change during nerve signal propagation is *not* approximately reversible, scientists do consider the claim that the temperature change during nerve signal propagation is approximately reversible true (since this is shown to be the case in several experiments).

That the truth of scientific claims is not determined relative to a single perspective is in line with Massimi’s account. And this is not the only respect in which the case study agrees with Massimi’s account. Scientists evaluate the truth of scientific claims based on certain standards of performance adequacy in the bio-electric perspective. One of the standards of performance adequacy that is used is accuracy with regard to Ohm’s law and Kirchhoff’s laws. Hodgkin and Huxley developed their equations for modelling the nerve signal by applying these laws to the electrical circuit with which they modeled the neural membrane. Another standard that is used is the empirical testability of the model. Scientists use electrical experiments on isolated squid axons to test how well the HH-model can predict key characteristics of the nerve signal. According to the scientists accepting the bio-electric perspective, the HH-model (in combination with the later identified ion channels) meets these two standards of performance adequacy. Therefore, true claims about nerve signals—including propagating nerve signals—can be made based on the ‘updated’ HH-model. However, scientists developing the thermodynamic perspective do not agree with this judgement. According to them, the constant membrane capacitance is not justified in the context of propagation experiments. As a result, this set of standards of performance adequacy is not met for *propagating* nerve signals in the bio-electric perspective. And, consequently, claims about propagating nerve signals cannot be considered cross-perspectivally true.

### Progress in explanatory understanding of nerve signal propagation within the thermodynamic perspective

In this section, we will demonstrate that scientists developing the thermodynamic perspective on nerve signal propagation aim to contribute to the progress in explanatory understanding of this phenomenon compared to the bio-electric perspective in two ways. First, by answering more what-if-things-had-been-different question about this phenomenon, which is in line with Saatsi’s account. Second, by exposing and criticizing the constant membrane capacitance assumption underlying the explanation of nerve signal propagation in the bio-electric perspective. As in Sect. 4.1, we will end this section by comparing the case study with the accounts of Massimi and Saatsi.

The scientists developing the thermodynamic perspective on nerve signal propagation accept and use evidence about this phenomenon that was produced in the twentieth century, whilst the bio-electric perspective was being developed. However, rather than taking the HH-model and the voltage clamp as a starting point, they focus on the electrical and non-electrical aspects associated with the nerve signal during propagation. According to these scientists, one should start from thermodynamic principles in order to deduce the expected behavior of nerve signal propagation. Thus, only explaining the electrical manifestation of the nerve impulse, as is done in the bio-electric perspective, is a non-starter. Both electrical and non-electrical manifestations follow from the laws of thermodynamics, so one cannot justifiably choose to explain one without considering the other. Moreover, scientists in the thermodynamic perspective (Appali et al., 2012; Heimburg & Jackson, 2006; Schneider, 2021) criticize the HH-model for its ad hoc parameters to fit the equations of the sodium and potassium conductance to experimental data. In addition, they point out that the assumption that a pulse exists which propagates with constant speed independent of voltage is also ad hoc. Due to these ad hoc assumptions and parameters the predictive power of the HH models becomes zero.^9^ As a consequence, in the thermodynamic perspective we see that different what-if questions about nerve signal propagation are addressed with the aim of increasing explanatory understanding of this phenomenon that cannot easily be obtained in the bio-electric perspective.^10^

Within this perspective, many experiments are done with artificial lipid membranes to enable the study of acoustic pulse propagation. Using such artificial lipid membranes, counterfactual dependencies have been established between changes in pressure, voltage and optical properties in membrane interfaces during acoustic pulse propagation (Griesbauer et al., 2012; Shrivastava & Schneider, 2013). Yet, these artificial lipid membranes do not contain ion channel proteins, which is an idealization compared to the neural membrane. This has been justified with the argument that the physics of the neural membrane interface is important for nerve signal propagation according to the thermodynamic theory, not molecular structure (Schneider, 2021). Moreover, it has been shown that these acoustic pulses have similar features as nerve signals: for instance, a bi-phasic pulse shape (Shrivastava & Schneider, 2014) and an all-or-none behavior (Shrivastava et al., 2015). In addition, it has been established that there is a counterfactual dependence between temperature and the heat capacity of both artificial and biological membranes (Heimburg & Jackson, 2005). Together, these experiments suggest that the lipid membrane plays a more active role during nerve signal propagation than just acting as a static barrier that contains ion channels who do all the work.

However, scientists developing the thermodynamic perspective on nerve signal propagation do not only try to increase explanatory understanding of nerve signal propagation by answering more and different what-if questions. They also reflect on the approach to gain explanatory understanding of this phenomenon in the bio-electric perspective and point out the limitations of this approach. In addition to criticizing the sole focus on electric manifestations of the nerve signal and ad hoc parameters in the HH-model, they have exposed the assumption regarding the constant membrane capacitance underlying the bio-electric perspective (Heimburg & Jackson, 2006), addressing a blind spot in the bio-electric perspective. Moreover, they have indicated why this assumption is problematic in nerve signal propagation experiments: the membrane capacitance cannot be assumed constant in this context due to the swelling of the neural membrane which can also account for the measured electrical manifestations of nerve signal propagation (Andersen et al., 2009). In addition, they have stressed that the bio-electric approach to nerve signal propagation cannot account for the temperature changes that have been measured during nerve signal propagation.^11^

So, these scientists show how the assumption that has enabled explanatory understanding in the bio-electric perspective hinders further progress in explanatory understanding. When scientists lose sight of this assumption and its possible consequences, they might assume they are able to explain nerve signal propagation and conclude that this phenomenon does not require further attention. However, even though the assumption has been highly successful and has led to an increase of explanatory understanding of nerve signal propagation, it also constrains the what-if questions that are asked about the phenomenon. With the thermodynamic perspective, a new approach (to the study of nerve signal propagation) is suggested, which leads to different what-if questions about the phenomenon. Questions that have been neglected in the bio-electric perspective, and that cannot be answered using the current methods and models employed in this perspective. To be more concrete, the thermodynamic approach opens up new pathways to increase explanatory understanding of nerve signal propagation by pointing out that the neural membrane may swell during nerve signal propagation, and that this may be an important contributing factor to the phenomenon of nerve signal propagation. Addressing and answering questions about this factor may contribute to capturing better the worldly dependency-relations regarding the non-electrical aspects of nerve signal propagation than can be currently achieved with the bio-electric perspective.

Thus, this case study suggests that progress in explanatory understanding of a phenomenon across perspectives is not only pursued by trying to answer different and more what-if-things-had-been-different questions about the phenomenon. In addition, this aim is pursued by criticizing the limitations of the acquired understanding due to the possibly non-factive aspects of the models underlying the other perspective. Scientists accepting a particular perspective may be unaware of these limitations, but they can become exposed using the different approach in the criticizing perspective. Of course, criticizing the possibly non-factive aspects of used models does not directly lead to an increase in the epistemic dimension of understanding by answering more what-if questions about the phenomenon of interest. It might even lead to a decrease in answered and answerable questions about the phenomenon, since some previously acceptable answers might be judged unjustified given the criticism from the other perspective. But it does contribute to the epistemic dimension of understanding by suggesting where to look for more answers regarding worldly dependency-relations about the phenomenon.

This is in line with Saatsi’s account that progress in explanatory understanding is related to capturing worldly dependency-relations better and better. However, realists should not be satisfied by capturing the current state of affairs regarding the questions that are already answered or answerable in science. In addition, they should be concerned with how worldly dependency-relations can be captured even better in the future. Looking at the case in hand, it turns out that criticizing the possibly non-factive aspects of models used provides a way to promote this goal. Thus, the value of interaction across perspectives lies not only in providing more answers to what-if questions about a phenomenon or in making the existing explanations of the phenomenon more cognitively salient for scientists due to the use of different models, but also in exposing the limitations of the understanding obtained. The latter is particularly important for stimulating interest in addressing more what-if questions that have been overlooked or purposefully neglected in the past. This shows that both the epistemic and the pragmatic dimension of understanding are important for the realist. The epistemic dimension because this dimension measures the *actually* answerable what-if questions about the phenomenon; the pragmatic dimension because this dimension measures the *potentially* answerable what-if questions about the phenomenon.

Moreover, our case study suggests that things are more complicated than Massimi suggests. On Massimi’s view, scientists in the context of assessment do not have to agree with the standards of performance adequacy adopted in the context of use in order to conclude that these standards have been met and cross-perspectival truth has been achieved. Although our case study does not deny this, it does show that Massimi’s view might prompt us to overlook the impact of different standards of performance adequacy across perspectives. Scientists in the thermodynamic perspective do not agree with the methodological choices that have been made in the bio-electric perspective: focusing solely on the electrical manifestations of nerve signal propagation is unjustifiable when thermodynamic laws are used as a starting point for inquiry, and so are the ad hoc parameters in the HH-model. This is a reason for scientists in the thermodynamic perspective to focus on aspects of nerve signal propagation that are overlooked in the bio-electric perspective using different methodological standards. This is in line with Saatsi’s view, since the thermodynamic approach will lead to answering different what-if questions about the phenomenon than the ones addressed in the bio-electric perspective. Yet, also Saatsi does not make the most of the different standards of performance adequacy that are used across perspectives. The different standards adopted in the thermodynamic perspective also lead to criticism of the approach adopted in the bio-electric perspective, pointing out what hinders progress in explanatory understanding within this perspective and suggesting new pathways for future research that may lead to further increase in understanding of the phenomenon of nerve signal propagation.

The case study also suggests why Saatsi’s view is more informative than Massimi’s view regarding another point. What if explanatory models in a context of use and a context of assessment share non-factive aspects without scientists being aware of their non-factivity? In that case, it can turn out that the two perspectives agree that a certain knowledge claim based on these explanatory models satisfies the standards of performance adequacy in the context of use. However, a third perspective might use another explanatory model that does not share these non-factive aspects and conclude that the knowledge claim does not meet the standards of performance adequacy. It seems that this implies that the knowledge claim cannot be considered cross-perspectivally true.

In such situations, Saatsi’s notion of explanatory understanding is more informative, since it can accommodate degrees of understanding. When two perspectives agree on certain explanations of a phenomenon, it can be concluded that there is a certain degree of understanding. If a third perspective disagrees, whilst showing that certain worldly dependency-relations are not captured well enough, it can be concluded that there is still understanding but of a lower degree than first supposed. Accordingly, this situation shows the additional value of criticizing the non-factive aspects of models used in other perspectives. It can show that the obtained understanding is not complete, even though this could have been supposed in the perspective. Of course, this does not mean that the perspective doesn’t provide any understanding of the phenomenon at all, but only that this understanding is more limited than believed before.

Of course, the perspectivist notion of truth might perhaps be adapted in order to accommodate degrees in truth across multiple perspectives. But we submit that it is more fruitful to adopt Saatsi’s approach and develop perspectivist accounts centered around explanatory understanding, since this notion can already accommodate degrees in understanding.

## Conclusion

Our case study provides a new insight for the debate on perspectivism. We have established that progress in explanatory understanding can be promoted by exposing the limitations of the acquired understanding of nerve signal propagation within the bio-electric perspective due to the possibly non-factive aspects that are needed to make the models in this perspective intelligible. Through the use of a different approach in the thermodynamic perspective it has become apparent that certain questions have been neglected in the bio-electric perspective that need to be addressed in order to determine whether worldly dependency-relations about the phenomenon of nerve signal propagation can be captured better than is currently the case. Moreover, informed by the thermodynamic perspective suggestions have been made where to look for these answers.

Using this insight from our case study, we have argued that in addition to the epistemic dimension of understanding, its pragmatic dimension is important for the realist. Although criticizing assumptions adopted in a particular perspective does not directly lead to an increase in explanatory understanding by *actually* answering more what-if questions about a phenomenon, it does show what *potentially* answerable what-if questions about the phenomenon have been overlooked or purposefully neglected thus far. Different perspectives are not just an inevitable result of our cognitive limitations, but also an asset to promote progress in explanatory understanding by exposing and criticizing the possibly non-factive aspects of rival perspectives.

Finally, although we do not have the space to discuss this in detail here, we would like to point out a general implication of our case study for interdisciplinary research. The debate about perspectivism has already been connected to the explanatory challenge for interdisciplinary research before (Fagan, 2019). Our case study suggests that interdisciplinary research would be well advised to aim at increasing explanatory understanding across the perspectives that are involved in the research. This can be achieved by answering more and different what-if-things-had-been-different questions about the phenomenon of interest using the models and tools of the different disciplines involved. But in addition, our case study suggests that it can be fruitful to scrutinize the possibly non-factive aspects of the approaches used in a particular perspective using the insights from another perspective. This can help to expose and, if necessary, criticize the assumptions underlying the perspectives and becoming aware of the limitations of the obtained explanatory understanding. Thus, guidance can be provided to address more what-if questions that have been overlooked and/or cannot be tackled yet as a result of assumptions underlying the perspectives. In this way, progress in explanatory understanding may be promoted substantially.

## Footnotes

1 The current in a capacitor ($${I}_{C})$$ is described by the following equation: $${I}_{C}= \frac{dQ}{dt}= \frac{d}{dt} \left(CV\right)=C\frac{dV}{dt}+V\frac{dC}{dt}$$, where $$Q$$ is charge, $$C$$ is capacitance and $$V$$ is voltage. By assuming $$C$$ to be constant, the last term in the equation becomes zero and one obtains the first term in Eq. 1 as a description of the capacity current.

2 Our discussion is based on reviews by Barchi (1988) and Trumpler (1997).

3 For more background information on this approach, see Drukarch et al. (2022).

4 The membrane capacitance has to be variable during the propagation of the acoustic pulse, since it is a function of the membrane area and thickness (which both change due to the density change associated with the acoustic pulse): ${C}_{M}={K}_{M}*{\upvarepsilon }_{0}*\frac{{A}_{M}}{{d}_{M}}$. In this equation, ${C}_{M}$ is the membrane capacitance, ${K}_{M}$ the dielectric constant of the membrane, ${\upvarepsilon }_{0}$ the permittivity of free space, ${A}_{M}$ the area of the membrane, and ${d}_{M}$ the membrane thickness.

5 Notice that the thermodynamic perspective on nerve signal propagation is still in the developmental stage. The relation between the thermodynamic theory and the biological phenomenon of nerve signal propagation has to be studied more extensively before it can be established that this perspective can provide an explanation of the phenomenon of nerve signal propagation that is overall more satisfactory than the explanation offered by the bio-electric perspective.

6 It might be debated whether this contrast is as sharp as Saatsi suggests. On the one hand, some philosophers, most famously Ryle (1949) and more recently Hyman (2015), challenge the standard conception of knowledge as a form of belief, arguing that it essentially involves abilities. On the other hand, authors such as Khalifa (2017) have argued that understanding can be reduced to knowledge, and even if one does not want to go that far, it seems plausible that understanding requires at least some knowledge, in addition to abilities. However, for the present discussion an initial distinction between knowledge as belief and understanding as ability suffices. We thank an anonymous reviewer for alerting us to this issue.

7 Hodgkin and Huxley collaborated with Katz for some of their experiments. They formulated the model together, though.

8 Without the voltage clamp, scientists would not have been able to study ionic currents during the action potential. And without the assumption that the membrane capacitance is constant in the HH-equations, it becomes impossible to separate capacity and ionic currents theoretically (see footnote 1).

9 Due to the ad hoc parameters and assumptions, no theory-guided falsifiable predictions can be formulated using the HH-model. The model only allows the formulation of theory-informed hypotheses for future investigation, which have no direct consequences for the correctness of the model itself. For a discussion of theory-guided versus theory-informed scientific practices, see Waters (2019).

10 We thank an anonymous reviewer for suggesting to explore differences in approaches to modelling in the different scientific communities.

11 Notice that the approach underlying the development of the thermodynamic perspective on nerve signal propagation also requires assumptions to make the models and theories used intelligible (an example is the use of artificial lipid membranes for studying acoustic pulse propagation). These assumptions can also become the subject of criticism.
